# Preoperative assessment of pleural adhesions in patients with lung cancer based on quantitative motion analysis with dynamic chest radiography: A retrospective study

**DOI:** 10.1002/acm2.14036

**Published:** 2023-05-17

**Authors:** Rie Tanaka, Isao Matsumoto, Tetsuya Takayama, Noriyuki Ohkura, Dai Inoue

**Affiliations:** ^1^ College of Medical Pharmaceutical & Health Sciences Kanazawa University Kanazawa Japan; ^2^ Department of Thoracic Surgery Kanazawa University Kanazawa Japan; ^3^ Department of Thoracic Surgery Kanazawa University Kanazawa Japan; ^4^ Department of Respiratory Medicine Kanazawa University Hospital Kanazawa Japan; ^5^ Department of Radiology Kanazawa University Hospital Kanazawa Japan

**Keywords:** digital radiography, lung cancer, motion analysis, pleural adhesions

## Abstract

**Purpose:**

Preoperative assessment of pleural adhesion is crucial for appropriate surgical planning. This study aimed to quantitatively evaluate the usefulness of motion analysis using dynamic chest radiography (DCR) for assessing pleural adhesions.

**Methods:**

Sequential chest radiographs of 146 lung cancer patients with or without pleural adhesions (n = 25/121) were obtained using a DCR system during respiration (registration number: 1729). The local motion vector was measured, and the percentage of poor motion area to the maximum expiration lung area (%lung area with poor motion) was calculated. Subsequently, percentage values ≥49.0% were considered to indicate pleural adhesions. Sensitivity, specificity, positive predictive value (PPV), and negative predictive value (NPV) were calculated to assess the prediction performance. The percentage of lung area with poor motion was compared between patients with and without pleural adhesions (*p* < 0.05).

**Results:**

DCR‐based motion analysis correctly predicted pleural adhesions in 21 out of 25 patients, with 47 false‐positive results (sensitivity, 84.0%; specificity, 61.2%; PPV, 30.9%; NPV, 94.9%). The lung with pleural adhesions showed a significantly greater %lung area with poor motion than the opposite lung in the same patient, similar to the cancerous lung in patients without pleural adhesions.

**Conclusion:**

On DCR‐based motion analysis, pleural adhesions could be indicated by an increase in the percentage of lung area with poor motion. Although the proposed method cannot identify the exact location of pleural adhesions, information regarding the presence or absence of pleural adhesions provided by DCR would help surgeons prepare for challenging surgeries and obtain informed consent from patients.

## 1 INTRODUCTION

Video‐assisted thoracoscopic surgery, a less invasive and well‐established approach for the surgical resection of lung cancer,^1^, ^2^, ^3^ is now commonly used as the first choice for many cases of lung cancer resection. However, the presence of pleural adhesions may increase the risk of lung injury from a trocar or scope, prolong the operation time, and lead to conversion to thoracotomy in severe cases.^4^ Therefore, the preoperative assessment of pleural adhesions is crucial for planning appropriate operative approaches.

Recent advances in medical imaging technologies have allowed the preoperative prediction of pleural adhesions in patients with lung cancer based on findings from four‐dimensional (4D) computed tomography (CT),^5^, ^6^, ^7^, ^8^ cine magnetic resonance imaging (MRI),^9^, ^10^, ^11^ and ultrasonography.^12^, ^13^, ^14^ These approaches assess pleural invasion and adhesion based on tumor motion during respiration relative to the adjacent lung structures. A lung tumor moving independently from the parietal or mediastinal pleura suggests that it does not invade or adhere strongly to the chest wall or pleura. Yamashiro et al.^7^ demonstrated that 4DCT had perfect diagnostic accuracy for pleural invasion/adhesion (Sensitivity: 100%, Specificity: 100%) compared to conventional chest CT (Sensitivity: 60%, Specificity: 77%). Similarly, Sakai et al.^9^ reported that the sensitivity, specificity, and accuracy of breathing dynamic cine MR in detecting chest wall invasion were 100%, 70%, and 76%, while those of conventional CT and MRI were 80%, 65%, and 68%, respectively. Regarding US approaches, a pooled analysis of 10 articles showed sensitivity and specificity of 71% and 96%, respectively.^12^ Despite the effectiveness of these approaches for preoperative prediction of pleural invasion and adhesion of lung tumors, they have some issues, such as the low availability of MRI and 4DCT and a limited field of view for ultrasonography. To address some of these limitations, dynamic chest radiography (DCR) has been proposed as a potential approach to assess pleural adhesions.

DCR is a flat‐panel detector (FPD)‐based functional X‐ray imaging technique that provides 15 sequential chest radiographs per second.^15^ Improvements in FPD technology have enabled quasi‐real‐time observation of both lungs with extremely low‐dose imaging; the total entrance surface dose can be less than the limit of two projections (1.9 mGy) recommended by the International Atomic Energy Agency.^16^, ^17^ Recent preliminary studies have demonstrated that DCR can detect pleural invasion or adhesion based on image findings,^18^, ^19^ such as localized reduction and distortion of lung motion, with a sensitivity of 88.0% and specificity of 83.5% at an average reading time of 20.4 min per case.^20^ However, the subjective assessment of moving lung structures on DCR requires knowledge of lung respiratory dynamics and extensive reading experience, as well as a long reading time. Thus, to allow the utilization of the DCR as a routine adhesion assessment, a qualitative analysis method for lung motion is essential. Although computer‐based approaches, such as optical flow and motion tracking techniques,^21^, ^22^ have been proposed, their diagnostic performance remains unevaluated in patients with cancer and pleural adhesions. This retrospective study aimed to quantitatively evaluate the usefulness of motion analysis for the assessment of pleural adhesions in patients with lung cancer with DCR. We conducted a study to evaluate the feasibility of identifying pleural adhesions by comparing quantified lung motion between affected and unaffected lungs.

## 2 METHODS

### 2.1 Participants

This study was a secondary analysis of the same clinical trial data from another study using a completely different analytical approach^20^ and had the same trial registration number as the primary trial (registration number: 1729). While the previous study was conducted using a subjective approach, this study used an objective approach. Therefore, the analytical methods and conclusions presented herein do not duplicate those of the previous report. All study participants provided informed consent, and the study design was approved by the institutional review board.

A total of 148 consecutive patients with lung cancer who visited Kanazawa University hospital between February 2016 and July 2019 were initially identified for this retrospective study. The study included patients who underwent both DCR and conventional chest CT as part of their routine clinical care before lung cancer surgery and those who underwent surgical removal of lung cancer. We excluded patients whose lung lesions were confirmed to be mesothelial tumors (*n* = 1) and those with an interval of more than 3 months between DCR and surgery (*n* = 1). Eventually, 146 preoperative patients (age, 40−88 years; mean age, 70 years; male‐to‐female ratio, 99:47) were assessed in this study. The presence or absence of pleural adhesions and invasion was retrospectively determined based on surgical videos, which were used as the ground truth. The findings were further classified into five grades according to the extent of invasion or adhesions on the lung surface: grade 0, no adhesions; grade 1, less than one‐third of the lung surface, no surgical disturbance; grade 2, one‐third to two‐third of the lung surface; grade 3, two‐third to < 1; and grade 4, adhesions on the entire lung surface. Pleural adhesions covering one‐third or more of the lung surface area may require longer operation times because of the need to separate adhesions. Therefore, 146 patients were further classified into invasion or adhesion‐negative (grades 0−1: *n* = 121) and invasion or adhesion‐positive (grades 2−4: *n* = 25) groups. The patient characteristics are summarized in Table 1. Twenty‐nine patients had tumors in the peripheral lung and/or adjacent to the chest wall (Grades 0−1: *n* = 26, Grades 2−4: *n* = 3), and none of the three patients with tumors and pleural adhesions had findings suggestive of pleural adhesions on conventional CT.

**TABLE 1:** Patient characteristics.

			Adhesion negative	Adhesion positive
Number			121	25
Age (years)			70.0 ± 9.5	71.0 ± 7.9
Sex (female/male)			44/77	3/22
Affected side	Right lung/left lung		81/39	17/8
	Others		1	0
Adhesion grade	Adhesion negative	Grade 0	92	
		Grade 1	29	
	Adhesion positive	Grade 2		13
		Grade 3		8
		Grade 4		4

### 2.2 Imaging procedures

**FIGURE 1:**
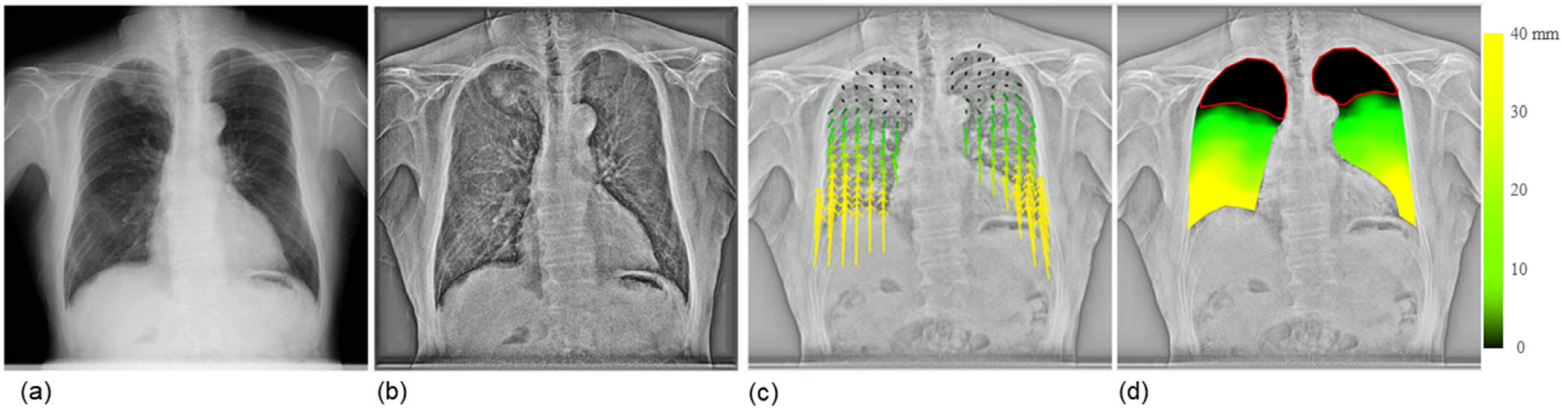
An example of a true‐negative case correctly predicted as not showing pleural adhesion by dynamic chest radiography (DCR)‐based quantitative motion analysis. Frame of the original DCR image (a), bone‐suppressed DCR with enhanced high spatial frequency (b), vector‐map images (c), and displacement‐map image (d) of a 72‐year‐old man with right lung cancer (grade 0). The patient showed poor motion in 27.5% and 28.5% of the right and left lung areas, respectively, a predicted VC of 129.6%, predicted FEV1 of 90.7%, FEV1/FVC ratio of 52.4, and predicted DLco of 52.4%. DLco, diffusing capacity of the lung for carbon monoxide; FEV1, forced expiratory volume in 1 s; FEV1/FVC, forced expiratory volume in 1 s/forced vital capacity; VC, vital capacity.

Sequential chest radiographs were obtained using a dynamic digital radiography system (Test Model; Konica Minolta, Tokyo, Japan) consisting of an indirect conversion FPD (PaxScan, 4343CB; Varex Imaging Corp., Utah, USA) and an X‐ray generator and tube (DHF‐155H II/UH‐6QC‐07E; Hitachi Healthcare, Ltd., Tokyo, Japan). Imaging was performed during forced breathing in the standing position and a posteroanterior direction, using pulsed irradiation of 15 frames/s (100 kV, 0.2 mAs/pulse, source‐to‐detector distance = 2.0 m).^15^ An automatic voice system was used for patient instruction to include one full respiratory cycle of forced breathing in approximately 14 s of imaging time (Figure 1a, Video 1a). The matrix and pixel sizes were 1024 × 1024 pixels and 417 × 417 μm^2^, respectively, with a 16‐bit grayscale. After selectively suppressing the shadows of bone structures in the lung region,^23^, ^24^ the high spatial frequency image components were enhanced to achieve selective motion tracking of lung structures, such as bronchi and blood vessels in the lungs (Figure 1b, Video 1b). The enhancement of high‐spatial frequency components was conducted based on the multiple resolution decomposition techniques; multiple images with different non‐sharpness were subtracted from the original image to decompose into multiple images with different frequency components.^25^ The images were then selectively combined so that the vascular shadows were emphasized.

### 2.3 Motion analysis of lung structures

Sequential images with the diaphragm moving upward were selected to measure the motion vector of lung structures in the expiratory phase. The Dense Optical Flow by Gunnar FarneBack technique^26^ was used to estimate pixel‐by‐pixel motion (optical flow) between two consecutive frames in each local lung area on bone‐suppressed images. Optical flow was calculated as an array of flow vectors in terms of magnitude and direction. The total amount of movement was calculated by connecting the movement between two consecutive frames. An open‐source computer vision library (OpenCV 3.4.3) was used. The resulting output is a two‐dimensional vector field, where each vector is a displacement vector that shows the movement of points from a reference frame to the current frame. In this study, the reference frame was set to the maximum inspiratory frame, that is, the first frame in the sequential images of the expiratory phase.

### 2.4 Visualization of the motion vector

To facilitate visual evaluation, the motion vector was visualized with arrows, indicating the orientation and displacement from the maximum inspiratory frame, as shown in Figure 1c and Video 1c (hereafter, vector‐map images). Pleural adhesions were observed as locally restricted and/or distorted motion findings in the lung structures.^20^ Therefore, we defined the local lung area with a displacement less than the threshold value as local lung areas with poor motion. In this study, the threshold value was set to 1.5 mm to account for errors in the motion analysis. The threshold value was determined on the basis of the receiver operating characteristic (ROC) analysis, where the area under the curve (AUC) was maximized with stable diagnostic performance. In addition, to account for the displacement attributable to the upward movement of the diaphragm during expiration, the horizontal component of the motion vector was ignored, and the ascending component was extracted. The downward component was treated as a negative value. The arrows were also expressed on a color scale, where the lung area with an upward movement was indicated by yellow (>40 mm), gradually changing to green (>10 mm), and the lung area with a displacement less than the threshold value was indicated by black (<1.5 mm). In addition, the local lung displacement was mapped onto the maximum expiratory frame according to the same color scaling, as shown in Figure 1d and Video 1d (hereafter, a displacement‐map image). The local area of the lung with poor motion was surrounded by a red line on a displacement‐mapimage.

### 2.5 Calculation of the lung area with poor motion

The percentage of the local lung area with a poor motion to the maximum expiration lung area (%lung area with poor motion) was used as an index to determine the presence or absence of pleural adhesions. Although the shape and volume of the left and right lungs are different, evaluation based on the percentage of the lung area with a poor motion can eliminate the difference. The %lung area with poor motion was calculated using the following formula:

$$\begin{array}{ccc} & & \%\mathrm{lung}\;\mathrm{area}\;\mathrm{with}\;\mathrm{poor}\;\mathrm{motion}\;\\ & & \;=\;\frac{\mathrm{The}\;\mathrm{local}\;\mathrm{lung}\;\mathrm{area}\;\mathrm{with}\;\mathrm{poor}\;\mathrm{motion}}{\mathrm{The}\;\mathrm{maximum}\;\mathrm{expiration}\;\mathrm{lung}\;\mathrm{area}} \end{array}$$

If the %lung area with poor motion was greater than the cut‐off value, the patient was considered to have pleural adhesions. The sensitivity should be rather high to avoid false negatives in patients with severe adhesions. Thus, the cut‐off value was determined using the Youden index^27^, ^28^ to have practical sensitivity and specificity values for the detection of pleural adhesions. In this study, we identified pleural adhesions, which are problematic in surgery, as a reduction in the “% area of lung in motion” compared to the contralateral lung of the same patient or the cancerous lung of a patient without pleural adhesions.

### 2.6 Statistical analysis

Statistical analysis of the groups with or without pleural adhesions was performed using R software (Ver 4.1.3^29^) with the Wilcoxon signed‐rank sum test to determine whether the %lung area with poor motion differed significantly between the lung with cancer and the opposite lung. In contrast, the Mann–​Whitney *U* test was used to test whether the differences between patients with and without pleural adhesions were significant. Statistical significance was set at *p* < 0.05. The diagnostic performance of DCR‐based motion analysis was evaluated in terms of sensitivity, specificity, positive predictive value (PPV), and negative predictive value(NPV).

## 3 RESULTS

**FIGURE 2:**
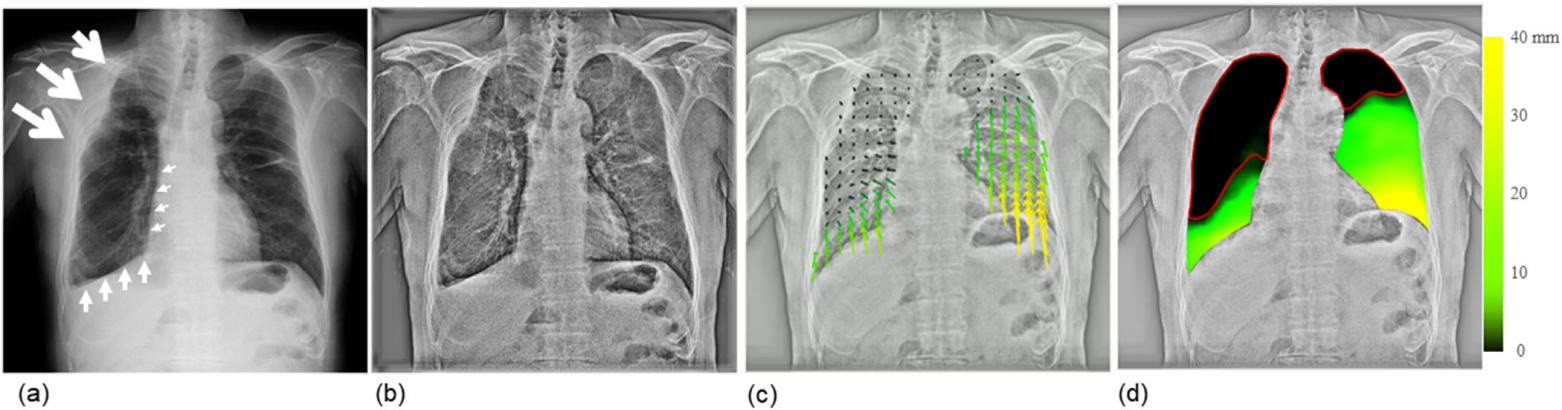
An example of a true‐positive case correctly evaluated as showing pleural adhesion by dynamic chest radiography (DCR)‐based quantitative motion analysis. Frame of the original DCR image (a), bone‐suppressed DCR with enhanced high spatial frequency (b), vector‐map images (c), and displacement‐map image (d) of a 69‐year‐old man with right lung cancer (grade 4 pleural adhesion over the right lung). The patient showed poor motion in 69.9% and 27.3% of the right and left lung areas, respectively, with a predicted VC of 73.9%, predicted FEV1 of 84.9%, FEV1/FVC ratio of 84.2, and predicted DLco of 55.3%. White arrows indicate areas with pleural invasion or adhesions; large, middle, and small arrows indicate invasion and strong and moderate adhesions, respectively. DLco, diffusing capacity of the lung for carbon monoxide; FEV1, forced expiratory volume in 1 s; FEV1/FVC, forced expiratory volume in 1 s/forced vital capacity; VC, vital capacity.

Vector analysis of DCR images quantified the respiratory motion of the lung structures. Figure 1 shows the results in a patient without pleural adhesions (a 72‐year‐old man with grade 0 right lung cancer). The resulting vector map quantitatively clarified the orientation and magnitude of the movement (Figure 1c, Video 1c). In general, patients without pleural adhesions showed right‐left symmetry and a top‐to‐down gradually decreasing distribution of local vectors, resulting in a displacement‐map image, as shown in Figure 1d. This patient had 27.5% and 28.5% lung areas with poor motion in the right and left lungs, respectively, and was correctly predicted to have no pleural adhesions. Figure 2 shows the results for a patient with pleural adhesions (a 69‐year‐old man with grade 4 right lung cancer). In this case, the lung cancer invaded the second to fifth ribs. In addition, the middle and lower lobes strongly adhered to the mediastinal and diaphragmatic surfaces. The resulting vector map indicated a right‐left asymmetric and widely distorted distribution of motion vectors, as shown in Figure 2c and Video 2c. The right lung with pleural adhesions indicated a larger poor motion area than did the left lung in a displacement‐map image, as shown in Figure 2d. This patient had 69.9% and 27.3% of lung areas with poor motion in the right and left lungs, respectively, and was correctly predicted to have pleural adhesions in the right lung.

**FIGURE 3:**
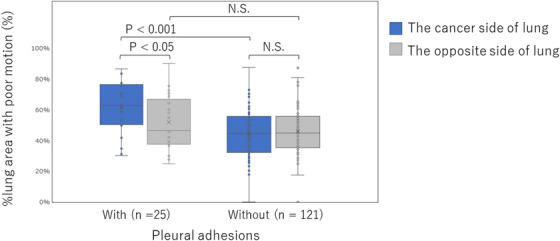
Box‐and‐whisker plots showing the percentage of lung area with poor motion to the maximum expiration lung area (%lung area with poor motion) on the lung with cancer and the opposite lung in patients with/without pleural adhesions. The error bar indicates the maximum value in the upper part and the minimum value in the lower part.

In patients with pleural adhesions (*n* = 25), the %lung area with a poor motion in the lung with cancer was significantly greater than that in the opposite lung (*p* < 0.05) (Figure 3). In contrast, in patients without pleural adhesions (*n* = 121), the %lung area with a poor motion did not differ significantly between the lungs with cancer and the opposite lung. In addition, the %lung area with poor motion on the cancerous side of the lung differed significantly between patients with and without pleural adhesions (*p* < 0.001) (Figure 3). However, the findings for the opposite lung did not show significant differences between the patients with and without pleural adhesions.

**FIGURE 4:**
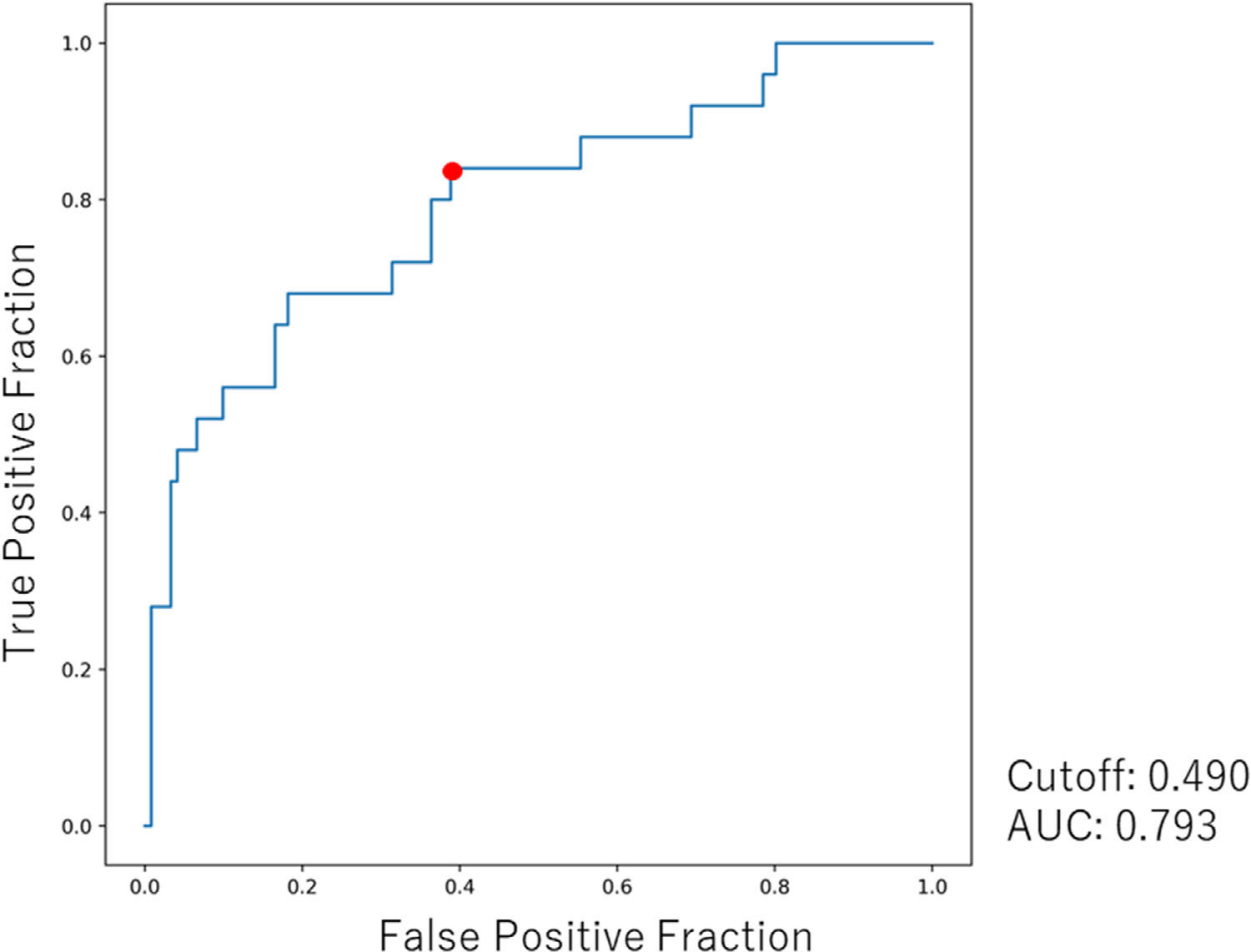
Receiver operating characteristic (ROC) curve for predicting pleural adhesions by DCR‐based quantitative motion analysis. The area under curve and cutoff value are 0.793 and 0.490, respectively.

Figure 4 shows the ROC curve for predicting pleural adhesions using the DCR‐based quantitative motion analysis. The AUC was 0.793 and the cut‐off value was 49.0% based on the Youden index. Table 2 shows the performance of DCR‐based quantitative motion analysis in pleural adhesion assessment for each adhesion grade. The present method correctly identified 21 out of 25 patients with pleural adhesions, including three patients with peripheral lung cancer who had no findings suggestive of pleural adhesions on conventional CT. The performance of DCR in detecting pleural adhesions had a sensitivity of 84.0%, specificity of 61.2%, PPV of 30.9%, and NPV of 94.9%.

**TABLE 2:** Diagnostic performance of dynamic chest radiography‐based quantitative motion analysis in each adhesion grade.

			Results of motion analysis	
Adhesion grade	Performance		Positive	Negative
Adhesion negative	Specificity			
Total (*n* = 121)	61.2%	(74/121)	47	74
Grade 0	59.8%	(55/92)	37	55
Grade 1	65.5%	(19/29)	10	19
Adhesion positive	Sensitivity			
Total (n = 25)	84.0%	(21/25)	21	4
Grade 2	92.3%	(12/13)	12	1
Grade 3	62.5%	(5/8)	5	3
Grade 4	100.0%	(4/4)	4	0

**FIGURE 5:**
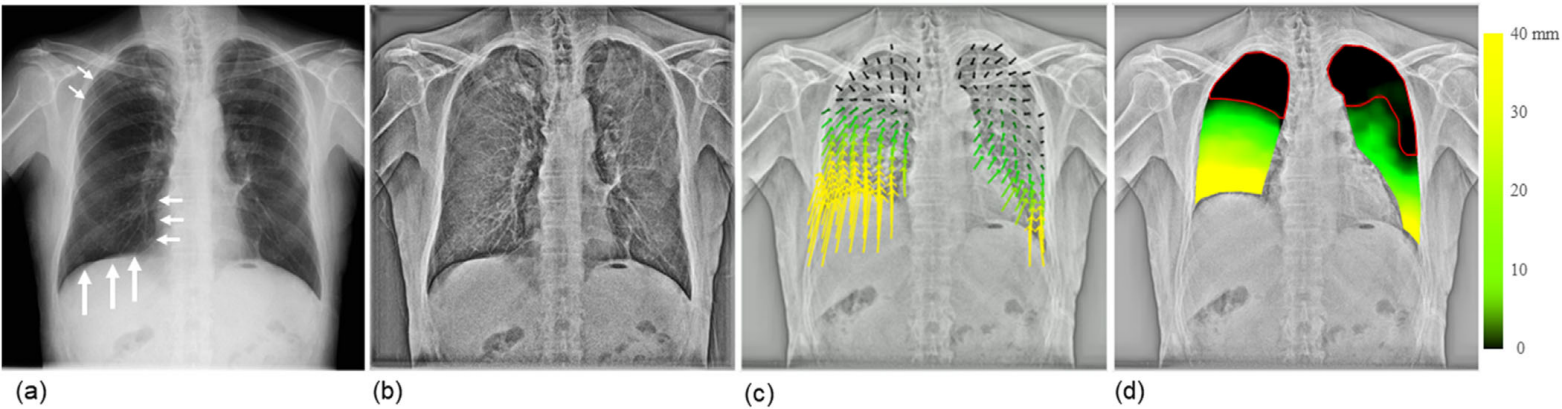
An example of a false‐negative case. Frame of the original dynamic chest radiography (DCR) images (a), bone‐suppressed DCR with enhanced high spatial frequency (b), vector‐map images (c), and displacement‐map image (d) of a 71‐year‐old man with right lung cancer (grade 2 pleural adhesion of the entire diaphragmatic surface). The patient showed poor motion in 34.4% and 45.9% of the right and left lung areas, respectively, a predicted VC of 90.5%, predicted FEV1 of 78.1%, FEV1/FVC ratio of 66.6, and predicted DLco of 41.6%. White arrows indicate areas with pleural adhesions; large, middle, and small arrows indicate strong, moderate, and mild adhesions, respectively. DLco, diffusing capacity of the lung for carbon monoxide; FEV1, forced expiratory volume in 1 s; FEV1/FVC, forced expiratory volume in 1 s/forced vital capacity; VC, vital capacity.

**FIGURE 6:**
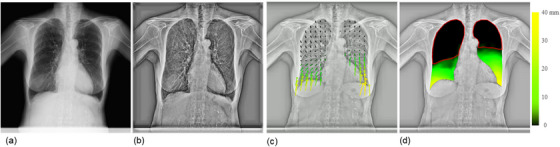
An example of a false‐positive case. Frame of the original dynamic chest radiography (DCR) images (a), bone‐suppressed DCR with enhanced high spatial frequency (b), vector‐map images (c), and displacement‐map image (d) of a 78‐year‐old female with right lung cancer (grade 0). The patient showed poor motion in 64.3% and 49.1% of the area of the right and left lungs, respectively; the predicted VC was 107.2%, predicted FEV1 was 124.0%, FEV1/FVC ratio was 69.2, and predicted DLco was 54.4%. DLco, diffusing capacity of the lung for carbon monoxide; FEV1, forced expiratory volume in 1 s; FEV1/FVC, forced expiratory volume in 1 s/forced vital capacity; VC, vital capacity.

Figure 5 and Video 3 show a false‐negative example of a 71‐year‐old man incorrectly predicted to have no pleural adhesions. In this case, the upper lobe (S2) slightly adhered to the dorsal and lateral thoracic surfaces. The middle and lower lobes strongly adhered to the diaphragmatic plane. Otherwise, the lateral thoracic region was free, resulting in only 34.4% of the area on the lung with cancer showing poor motion. Figure 6 and Video 4 show a false‐positive example of a 78‐year‐old woman with incorrectly predicted pleural adhesions. This patient had emphysema with thickening of the bronchial wall and insufficient respiration during imaging, resulting in 64.3% of the lung area with cancer showing poor motion.

**VIDEO 1:** An example of a true‐negative case (72‐year‐old man with grade 0 right lung cancer). The video shows the original dynamic chest radiography (DCR) images (a), bone‐suppressed DCR with enhanced high spatial frequency (b), vector‐map images (c), where the arrow length represents the movement from the first frame, and a displacement‐map image (d), where larger motion is indicated in yellow, gradually changing to green, while the lung area with poor motion is indicated in black.

**VIDEO 2:** An example of a true‐positive case (a 69‐year‐old man with right lung cancer and grade 4 pleural adhesion). The video shows the original dynamic chest radiography (DCR) images (a), bone‐suppressed DCR with enhanced high spatial frequency (b), vector‐map images (c), where the arrow length represents the movement from the first frame, and a displacement‐map image (d), where larger motion is indicated in yellow, gradually changing to green, while the lung area with poor motion is indicated in black.

**VIDEO 3:** An example of a false‐negative case (a 71‐year‐old man with right lung cancer and grade 2 pleural adhesion of the entire diaphragmatic surface). The video shows the original dynamic chest radiography (DCR) images (a), bone‐suppressed DCR with enhanced high spatial frequency (b), vector‐map images (c), where the arrow length represents the movement from the first frame, and a displacement‐map image (d), where larger motion is indicated in yellow, gradually changing to green, while the lung area with poor motion is indicated in black.

**VIDEO 4:** An example of a false‐positive case (a 78‐year‐old woman with grade 0 right lung cancer). The video shows the original dynamic chest radiography (DCR) images (a), bone‐suppressed DCR with enhanced high spatial frequency (b), vector‐map images (c), where the arrow length represents the movement from the first frame, and a displacement‐map image (d), where greater motion is indicated in yellow, gradually changing to green, while the lung area with poor motion is indicated in black.

## 4 DISCUSSION

We analyzed the DCR findings of 146 patients with or without pleural adhesions (*n* = 25/121) and confirmed that poor lung motion due to pleural adhesions could be quantified by DCR‐based motion analysis; lungs with pleural adhesions showed a significant increase in the %lung area with a poor motion than did the opposite lung in the same patient, similar to the findings for the lung with cancer in patients without pleural adhesions. DCR‐based motion analysis correctly identified 21 out of 25 patients with pleural adhesions based on an increase in %lung area with poor motion and yielded a specificity of 61.2% (74/121).

Previous studies have reported that lung structures show restricted and/or distorted motion in DCR due to restricted lung sliding in patients with pleural adhesions and smooth and left‐right symmetrical motions according to respiration in patients without adhesions.^20^ This study showed no significant difference in the %lung area with poor motion between the left and right sides in patients without pleural adhesions. In contrast, in patients with pleural adhesions, the %lung area with a poor motion was significantly greater in the lungs with pleural adhesions than in those without (*p* < 0.05). In a comparison of lungs with cancer, the %lung area with a poor motion in patients with pleural adhesions was significantly greater than in those without (*p* < 0.001). These results suggest that pleural adhesions could be predicted as an increase in the %lung area with poor motion on the DCR motion analysis.

Nevertheless, despite the use of optimal parameters in the DCR motion analyses, 4 and 47 cases showed false‐negative and false‐positive results, respectively. False‐negative diagnoses may occur because the %lung area with a poor motion did not increase when pleural adhesions were localized just above the diaphragm or at the lung apex. In cases with pleural adhesions over the entire diaphragm, as in Figure 5d, pleural adhesions may appear to be absent because the two structures move together. In contrast, insufficient respiration and/or underlying pulmonary disease can make pleural adhesion assessments based on lung motion challenging, which was also observed in radiologists’ readings.^20^ As shown in Figure 6, patients with chronic obstructive pulmonary disease and interstitial pneumonitis associated with decreased pulmonary function are likely to show the restricted motion of the entire lung. DCR‐based motion analysis showed one additional false‐negative case compared to radiologists’ readings, indicating that the performance of DCR‐based motion analysis in detecting pleural adhesions (sensitivity, 84.0%; specificity, 61.2%; PPV, 30.9%; NPV, 94.9%) was lower than that of radiologists’ readings (sensitivity, 88.0%; specificity, 83.5%; PPV, 52.4%; NPV, 97.12%).^20^ This performance degradation may have occurred because our computational algorithm detected pleural adhesions as an increase in %lung area with a poor motion, making it challenging to identify localized abnormal motion.

In relation to these findings, our study had several limitations. First, the %lung area with poor motion does not directly represent the location of adhesions. Thus, lung motion is reduced around adhesions, resulting in an increase in the %lung area with a poor motion in the lung. Therefore, the development of adhesion detection algorithms that focus on motion analysis of each local lung area is essential. The sensitivity of the proposed method to detect pleural adhesions could be improved by calculating the %lung area with poor motion for each lung area. In addition, comprehensive image interpretation combining image findings and quantified lung motion, such as displacement‐map images and the %lung area with a poor motion, is recommended to predict the location of pleural adhesions by DCR. Combining DCR with conventional CT or MRI may provide another potential solution for more accurate assessment of pleural adhesions in patients with lung cancer. Second, an insufficient inspired volume or underlying disease can cause poor motion of lung structures, even in the absence of adhesions. Thus, detection sensitivity should be increased by repeatedly practicing breathing techniques before imaging. Third, cases with the same degree of adhesions on both sides might show no difference in the %lung area with a poor motion in both lungs. If the %lung area with a poor motion is greater than that of the normal lung, it might be picked up aggressively. This is because, false positives, along with false negatives caused by moderate adhesions, are rarely surgically problematic. Thus, the sensitivity should be rather high to avoid false negatives in patients with severe adhesions, such as in patients undergoing lower lobectomies with severe diaphragmatic adhesions that might lead to an unexpected prolongation of the operative time. For clinical implementation, however, additional algorithms must be developed to reduce the increased false positives, while maintaining performance levels similar to radiologists’ readings.

Computer‐based motion analysis offers several advantages over a radiologist's reading. A previous study based on subjective evaluations^20^ reported three discordant cases in image interpretation of 146 cases between the two radiologists. With computerized approaches based on the %lung area with a poor motion, the evaluation is reproducible with no discordant cases. DCR can also detect local pulmonary impairments based on reduced respiration‐induced changes in lung density.^15^ Thus, computerized analysis of lung motion combined with changes in lung density has the potential to provide new evaluation metrics for pleural adhesions. Furthermore, analyzing the motion of the left and right lung based on imaging has the potential to improve the accuracy of pulmonary assessment. In addition, DCR‐based motion analysis was able to correctly identify pleural adhesions in peripheral lung cancers, whereas conventional CT did not show findings suggestive of pleural adhesions. The DCR system became commercially available in 2018 and was approved by the U.S. Food and Drug Administration in 2019. DCR can be performed as an additional examination in conventional radiography protocols. Although the patients were exposed to radiation during the examination, the total irradiation was approximately twice that of a conventional chest radiograph. Thus, DCR is acceptable because of its high information yield and rapid and simple imaging procedures in daily clinical settings. The present study demonstrated that DCR‐based motion analysis correctly identified 21 out of 25 patients with pleural adhesions based on an increase in the %lung area with poor motion and yielded a specificity of 61.2% (74/121). Although some issues need to be addressed in its clinical implementation, the feasibility of DCR‐based quantitative motion analysis for the preoperative evaluation of pleural adhesions was ascertained in this study.

## 5 CONCLUSION

This retrospective study revealed that pleural adhesions could be predicted by DCR‐based quantitative motion analysis as an increase in the %lung area with a poor motion. Although the proposed method cannot identify the exact location of pleural adhesions, it may be useful to determine the presence or absence of surgically problematic pleural adhesions by comparing them between lungs. It may also help surgeons prepare for difficult surgeries and obtain informed consent from the patients. Further development of computerized methods to locally assess lung motion will pave the way for the routine preoperative evaluation of pleural adhesions using DCR.

## AUTHOR CONTRIBUTIONS

All authors made substantial contributions to all of the following: (1) the conception and design of the study, or acquisition of data, or analysis and interpretation of data, (2) drafting the article or revising it critically for important intellectual content, (3) final approval of the version to be submitted.

## CONFLICT OF INTEREST STATEMENT

This research was partially supported by Konica Minolta, Inc. (Tokyo, Japan). Konica Minolta had no role in the design, analyses, or reporting of this study.

## References

[1] McKenna RH , Houck W , Fuller CB . Video‐assisted thoracic surgery lobectomy: experience with 1,100 cases. Ann Thorac Surg. 2006;81:421‐425. 16427825 10.1016/j.athoracsur.2005.07.078

[2] Committee for Scientific Affairs, The Japanese Association for Thoracic Surgery , Shimizu H , Okada M , Toh Y , et al, Committee for Scientific Affairs, The Japanese Association for Thoracic Surgery . Thoracic and cardiovascular surgeries in Japan during 2018: annual report by the Japanese Association for Thoracic Surgery. Gen Thorac Cardiovasc Surg. 2021;69:179‐212. 33090365 10.1007/s11748-020-01460-w PMC7788037

[3] Yan TD , Black D , Bannon PG , McCaughan BC . Systematic review and meta‐analysis of randomized and nonrandomized trials on safety and efficacy of video‐assisted thoracic surgery lobectomy for early‐stage non‐small‐cell lung cancer. J Clin Oncol. 2009;7:2553‐2562. 10.1200/JCO.2008.18.2733 19289625

[4] Marty‐Ane CH , Canaud L , Solovei L , Alric P , Berthet JP . Video‐assisted thoracoscopic lobectomy: an unavoidable trend? A retrospective single‐institution series of 410 cases. Interact Cardiovasc Thorac. 2013;17:36‐43. 10.1093/icvts/ivt146 PMC3686401 23592725

[5] Sakuma K , Yamashiro T , Moriya H , Murayama S , Ito H . Parietal pleural invasion/adhesion of subpleural lung cancer: quantitative 4‐dimensional CT analysis using dynamic‐ventilatory scanning. Eur J Radiol. 2017;87:36‐44. 28065373 10.1016/j.ejrad.2016.12.004

[6] Yamashiro T , Moriya H , Tsubakimoto M , Nagatani Y , Kimoto T , Murayama S , investigators of ACTIve study group . Preoperative assessment of parietal pleural invasion/adhesion of subpleural lung cancer: advantage of software‐assisted analysis of 4‐dimensional dynamic‐ventilation computed tomography. Eur Radiol. 2019;29:5247‐5252. 30915563 10.1007/s00330-019-06131-w

[7] Nagatani Y , Hashimoto M , Oshio Y , et al, investigators of ACTIve study group . Preoperative assessment of localized pleural adhesion: utility of software‐assisted analysis on dynamic‐ventilation computed tomography. Eur J Radiol. 2020;133:109347. 33166835 10.1016/j.ejrad.2020.109347

[8] Hashimoto M , Nagatani Y , Oshio Y , et al, investigators of ACTIve study group . Preoperative assessment of pleural adhesion by four‐dimensional ultra‐low‐dose computed tomography (4D‐ULDCT) with adaptive iterative dose reduction using three‐dimensional processing (AIDR‐3D). Eur J Radiol. 2018;98:179‐186. 29279160 10.1016/j.ejrad.2017.11.011

[9] Sakai S , Murayama S , Murakami J , Hashiguchi N , Masuda K . Bronchogenic carcinoma invasion of the chest wall: evaluation with dynamic cine MRI during breathing. J Comput Assist Tomogr. 1997;21:595‐600. 9216765 10.1097/00004728-199707000-00013

[10] Shiotani S , Sugimura K , Sugihara M , et al. Diagnosis of chest wall invasion by lung cancer: useful criteria for exclusion of the possibility of chest wall invasion with MR imaging. Radiat Med. 2000;18:283‐290. 11128398

[11] Akata S , Kajiwara N , Park J , et al. Evaluation of chest wall invasion by lung cancer using respiratory dynamic MRI. J Med Imaging Radiat Oncol. 2008;52:36‐39. 18373824 10.1111/j.1440-1673.2007.01908.x

[12] Cassabelli N , Caroli G , Dolci G , et al. Accuracy of transthoracic ultrasound for the dictation of pleural adhesions. Eur J Cardiothorac Surg. 2012;42:813‐818. 22518039 10.1093/ejcts/ezs144

[13] Wei B , Wang T , Jiang F , Wang H . Use of transthoracic ultrasound to presict pleural adhesions: a prospective blinded study. Thorac Cardiovasc Surg. 2012;60:101‐814. 21442577 10.1055/s-0030-1270760

[14] Bandi V , Lunn W , Ernst A , Eberhardt R , Hoffmann H , Herth F . Ultrasound vs. CT in detecting chest wall invasion by tumor: a prospective study. Chest. 2007;133:881886. 10.1378/chest.07-1656 17951616

[15] Tanaka R . Dynamic chest radiography: flat‐panel detector (FPD) based functional X‐ray imaging. Radiol Phys Technol. 2016;9:139‐153. 27294264 10.1007/s12194-016-0361-6

[16] International Basic Safety Standards for Protection Against Ionizing Radiation and for the Safety of Radiation Sources. IAEA Safety Series No. 115. Vienna: International Atomic Energy Agency (IAEA); 1996;279.

[17] Annex IV , Schedule III . Guidance levels of dose, dose rate and activity for medical exposure. Radiological Protection for Medical Exposure to Ionizing Radiation. Safety Guide. IAEA Safety Standards Series No. SSG‐46. Vienna: International Atomic Energy Agency (IAEA); 2018.

[18] Tamura M , Matsumoto I , Saito D , et al. Dynamic chest radiography: novel and less‐invasive imaging approach for preoperative assessments of pleural invasion and adhesion. Radiol Case Rep. 2020;15:702‐704. 32280402 10.1016/j.radcr.2020.02.019 PMC7138928

[19] Tamura M , Matsumoto I , Saito D , Yoshida S , Takata M , Takemura H . Case report: uniportal video‐assisted thoracoscopic resection of a solitary fibrous tumor preoperatively predicted visceral pleura origin using dynamic chest radiography. J Cardiothorac Surg. 2020;15:166. 32641164 10.1186/s13019-020-01212-0 PMC7346465

[20] Tanaka R , Inoue D , Izumozaki A , Takata M , Tamura M , Matsumoto I . Preoperative evaluation of pleural adhesions with dynamic chest radiography: a retrospective study of 146 patients with lung cancer. Clin Radiol. 2022;77:e689‐e696. 35778295 10.1016/j.crad.2022.05.016

[21] Tanaka R , Sanada S , Suzuki M , Kobayashi T , Matsui T , Inoue H . Breathing chest radiography using a dynamic flat‐panel detector combined with computer analysis. Med Phys. 2004;31:2254‐2262. 15377092 10.1118/1.1769351

[22] Tanaka R , Samei E , Segars WP , et al. Assessment of pleural invasion and adhesion of lung tumors with dynamic chest radiography: a virtual clinical imaging study. Med Phys. 2021;48:1616‐1623. 33533481 10.1002/mp.14750 PMC8587535

[23] Miyoshi T , Yoshida J , Aramaki N , et al. Effectiveness of bone suppression imaging in the detection of lung nodules on chest radiographs: relevance to anatomic location and observer's experience. J Thorac Imaging. 2017;32:398‐405. 28914743 10.1097/RTI.0000000000000299

[24] Kodama N , Loc TV , Hai PT , et al. Effectiveness of bone suppression imaging in the diagnosis of tuberculosis from chest radiographs in Vietnam: an observer study. Clin Imaging. 2018;51:196‐201. 29860192 10.1016/j.clinimag.2018.05.021

[25] Mallat SG . A theory for multiresolution signal decomposition: the wavelet representation. IEEE Trans Pattern Anal Mach Intell. 1989:674‐693.

[26] Farnebäck G . Two‐frame motion estimation based on polynomial expansion. In: Bigun J , Gustavsson T , eds. Image Analysis. SCIA 2003.Lecture Notes in Computer Science. Springer. https://docs.opencv.org/3.4.3/de/d9e/classcv_1_1FarnebackOpticalFlow.html

[27] Youden WJ . Index for rating diagnostic tests. Cancer. 1950;3:32‐35. 15405679 10.1002/1097-0142(1950)3:1<32::aid-cncr2820030106>3.0.co;2-3

[28] Fluss R , Faraggi D , Reiser B . Estimation of the Youden Index and its associated cutoff point. Biom J. 2005;47:458‐472. 16161804 10.1002/bimj.200410135

[29] R Development Core Team (2020). R: A Language and Environment for Statistical Computing. R Foundation for Statistical Computing; 2020.

